# Stabilization of Beeswax-In-Water Dispersions Using Anionic Cellulose Nanofibers and Their Application in Paper Coating

**DOI:** 10.3390/nano13162353

**Published:** 2023-08-16

**Authors:** Genís Bayés, Roberto J. Aguado, Quim Tarrés, Jaume Planella, Marc Delgado-Aguilar

**Affiliations:** 1 LEPAMAP-PRODIS Research Group, University of Girona, Maria Aurèlia Capmany, 61, 17003 Girona, Spain; gbayes@noel.com (G.B.); joaquimagusti.tarres@udg.edu (Q.T.); m.delgado@udg.edu (M.D.-A.); 2 Noel Alimentària SAU, Pla de Begudà, 17857 Begudà, Spain; jplanella@noes.es

**Keywords:** barrier properties, beeswax, cellulose nanofibers, hydrophobic coating, nanocellulose, paper, Pickering emulsions, TEMPO-mediated oxidation

## Abstract

Beeswax is a bio-sourced, renewable, and even edible material that stands as a convincing option to provide paper-based food packaging with moisture resistance. Nonetheless, the difficulty of dispersing it in water limits its applicability. This work uses oxidized, negatively charged cellulose nanofibers along with glycerol to stabilize beeswax-in-water emulsions above the melting point of the wax. The synergistic effects of nanocellulose and glycerol granted the stability of the dispersion even when it cooled down, but only if the concentration of nanofibers was high enough. This required concentration (0.6–0.9 wt%) depended on the degree of oxidation of the cellulose nanofibers. Rheological hindrance was essential to prevent the buoyancy of beeswax particles, while the presence of glycerol prevented excessive aggregation. The mixtures had yield stress and showed pseudoplastic behavior at a high enough shear rate, with their apparent viscosity being positively influenced by the surface charge density of the nanofibers. When applied to packaging paper, the nanocellulose-stabilized beeswax suspensions not only enhanced its barrier properties towards liquid water (reaching a contact angle of 96°) and water vapor (<100 g m^−2^ d^−1^), but also to grease (Kit rating: 5) and airflow (>1400 Gurley s). While falling short of polyethylene-coated paper, this overall improvement, attained using only one layer of a biobased coating suspension, should be understood as a step towards replacing synthetic waxes and plastic laminates.

## 1. Introduction

Cellulose nanofibers (CNFs) can simultaneously fulfill the roles of rheology modifiers and Pickering stabilizers in oil-in-water emulsions, especially if they hold a negative charge [1]. On the one hand, their interactions with water molecules grant a large hydration shell, hindering the translational movement of the molecules with respect to the nanofibers. This partial trapping of water, along with a high aspect ratio, is translated into a higher viscosity and yield stress [2,3]. On the other hand, according to the DLVO theory, the electrostatic repulsion between the nanofibers that are adsorbed to the dispersed phase of an oil-in-water emulsion provides a potential energy barrier that prevents coalescence of oil droplets [4,5].

One of the most popular ways to produce anionic CNFs involves the regioselective oxidation of primary hydroxyl groups of cellulose to carboxylate groups, usually attained using 2,2,6,6-tetramethylpiperidine 1-oxyl radical (TEMPO), sodium hypochlorite, and a bromide salt [6,7]. This is generally followed by a disruptive mechanical treatment [6,8]. The resulting TEMPO-oxidized cellulose nanofibers (TOCNFs) are typically 5–50 nm in diameter and 0.3–3 µm in length. Their carboxyl group content mainly depends on the amount of hypochlorite used, reaching a maximum of roughly 1.5 mmol/g [9,10]. Regarding the aforementioned application as a thickener, aqueous suspensions of TOCNFs may attain gel behavior, along with yield stress values above 20 Pa, at concentrations as low as ~0.3 wt% [3,11]. This, in turn, is also useful for the Pickering stabilization of oil-in-water systems, since one of the mechanisms postulated for this task is the rheological hindrance of the motion of the droplets [1].

In the literature, colloidal sols where the dispersed phase is a wax, despite being solid at room temperature, are also generally regarded as oil-in-water emulsions [12,13]. It could be said that “wax emulsion” is a common [12,14], yet arguable, naming for wax-in-water dispersions at any temperature. Since wax particles are solid at standard temperatures and pressures, speaking in terms of emulsions may be inaccurate. Nonetheless, the strategy of stabilization generally incorporates all agents when the wax is molten [14,15]; i.e., this stabilization is indeed an emulsification.

Natural waxes are intrinsically hydrophobic materials with a high potential to replace polyethylene-based waxes, paraffin wax, acrylic waxes, polyoxyethylene glycol waxes, styrene-butadiene latex, and other synthetic substances that, despite their proven usability, ultimately come from fossil resources [16]. Beeswax (BW), like other natural waxes, has some key advantages that may convince manufacturers to use it in water-resistant products. First, unless it is abusively exploited, it is a renewable resource. Second, it can be biodegraded by some worms and other organisms with proper hydrolytic enzymes [17]. Third, despite its biodegradability, BW displays antimicrobial activity towards some of the most worrisome foodborne pathogens [18]. Fourth, it is considered edible [19], although only in small amounts. Fifth, it has great resistance to chemicals (including acids, alkalis, and organic solvents) and ultraviolet radiation [20].

However, the difficulty of dispersing BW in aqueous media, even in the presence of typical surfactants or co-solvents, limits its applicability. One of the suggested uses that is affected by such limitations is paper coatings. Paper, a bio-sourced, renewable, and biodegradable material, seems to stand as a promising candidate to replace single-use plastics in food packaging [21,22]. However, the surface of paper is hydrophilic, while many food packaging applications strictly demand moisture resistance. BW-based coatings, as opposed to plastic laminates, synthetic latex, and synthetic waxes, appeal to many researchers when it comes to making paper competitive with common plastics in terms of its barrier properties [14,19]. Table 1 displays some advances in this sense, also using nanocellulose or other polysaccharides to stabilize BW in water. The resulting hydrophobic, bio-based materials comprise films, edible coatings to wrap foodstuffs, and paper.

This work assesses the properties and the convenience of using BW-in-water emulsions following synergetic stabilization with TOCNFs and glycerol. This assessment began with a macroscopic approach, seeking to determine the critical concentration of TOCNFs with different degrees of oxidation to attain homogeneity. Furthermore, the stabilized BW particles and BW-coated paper were visualized using microscopy. The rheological properties of the representative dispersions were measured under different temperatures, shear rates, and TOCNF concentrations. Based on previous observations, certain systems were used for paper coating, and the improvement that they exerted on the barrier properties was assessed.

## 2. Materials and Methods

### 2.1. Materials

The cellulose source chosen for nanocellulose production was a bleached eucalyptus kraft pulp (BEKP) from Ence Celulosa y Energía, S.A. (Navia, Spain). TEMPO, heptane, toluene, and castor oil were purchased from Sigma-Aldrich (Schnelldorf, Germany. NaClO (10%, *w/v*), NaBr, and NaOH were purchased from Sharlab (Sentmenat, Spain). BW pellets labeled “100% pure” were bought from Promora.

Kraft paper of food packaging grade with an approximate grammage of 156 g m^−2^ was provided by Billerud AB (Solna, Sweden). This kind of paper generally undergoes plastic lamination before becoming commercially available as food packaging, but this work replaced this step with BW-based coatings.

### 2.2. Production of TEMPO-Oxidized Cellulose Nanofibers

Preliminary experiments using unmodified, purely mechanical, nominally neutral micro-/nanofibers resulted in a high difficulty to obtain durable macroscopic homogeneity. To imparting electrostatic repulsion, which helps prevent coalescence in accordance with the DLVO theory [4,25], cellulose was oxidized before nanofibrillation to acquire a negative surface charge.

A total of 30 g of BEKP (on a dry weight basis) was suspended in water and stirred at 3000 rpm for 10 min using a pulp disintegrator from IDM (Gipuzkoa, Spain) that complied with ISO standard 5263. The pulp was divided into three batches (10 g each, dry-weight basis), and each batch was mixed with NaBr (1.00 g) and TEMPO (0.16 g). Afterwards, 5, 10, or 15 mmol of NaClO was added per gram of dry pulp, and the resulting oxidized batches were labeled “TOC-5”, “TOC-10”, and “TOC-15”, respectively. The regioselective oxidation took place at a 1 wt.% consistency under agitation with a 3-blade mechanical stirrer at approximately 400 rpm at 23 °C. A 0.5 M NaOH solution was added throughout the process to maintain the pH within the range of 10–10.5. The oxidation was considered finished once the pH stopped dropping below 10. The oxidized pulps were thoroughly washed with distilled water, vacuum-filtered, and diluted to a 1.5 wt.% consistency.

Fibrillation of the nanofibers was performed using an NS1001L2K high-pressure homogenizer (HPH) from GEA Niro Soavi (Parma, Italy). The TOC-5, TOC-10, and TOC-15 batches were passed 3 times at 300 bar, 3 times at 600 bar, and 3 times at 900, respectively. The batches were then stored in PET bottles at 4 °C. The properties of these TOCNFs, with relevance in terms of their electrostatic repulsion, are displayed in Table 2. The characterization techniques are described in previous works [10].

### 2.3. Preparation and Visual Inspection of BW-In-Water Emulsions

For each experiment, 20 g of BW pellets were mixed with 2 g of glycerol and a TOCNF slurry, so that the concentration of the latter was 1–10 g/kg based on the oven-dried weight. Water was added to bring the total weight to 200 g. The heterogeneous mixture was heated to 70 °C and agitated using a 3-blade overhead stirrer set at roughly 400 rpm to melt the BW. The system was then homogenized using an UltraTurrax T25 device from IKA (Staufen, Germany) at 12,000 rpm for 4 min.

The emulsions were stored undisturbed and covered at 23 °C for 22–24 h. After this time, they were visually inspected at 23 °C to locate the oil/serum interface (if any) and to discern whether the BW particles were visible to the naked eye, if macroscopic homogeneity had been attained, and to identify the creaming processes that might threat the stability of the suspension. Photographs of the emulsions were taken using a LED-illuminated light box (20 W, white, color temperature 6000 K, surface luminance 310 cd m^−2^).

### 2.4. Analysis of BW/TOCNF/Glycerol/Water Systems

Certain macroscopically homogeneous samples were subjected to optical microscopy using a DMR-XA microscope from Leica (Wetzlar, Germany). The modes of observation included halogen illumination and polarized light over a dark field. Then, the particle size was analyzed using the open-source software ImageJ (Version 1.53), as described elsewhere [6], but without using the Fractal Box Count plugin. Instead, the average between the maximum Feret’s diameter and the minimum Feret’s diameter, readily available in the native software package, was taken to indicate the particle size (d). OriginLab’s OriginPro 8.5 was then used to fit the resulting size distributions.

The viscosity of the stable emulsions, including different degrees of oxidation and concentrations of TOCNFs, was measured using a rheometer with concentric cylinder geometry from PCI (Albacete, Spain), model RVI-2. The radii of the spindle and the outer cylinder were 4 mm and 5 mm, respectively. The rheological behavior of the TOCNF-stabilized BW-in-water emulsions was compared with that of the aqueous TOCNF suspensions at the same consistency. The effects of temperature and the influence of the shear rate were studied.

### 2.5. Coating and Visualization of Paper

27 cm × 20 cm-sized food-grade paper sheets were immobilized using a K Control Coater from RK Print Coat Instruments (Litlington, UK). For the coating, two concentrations of TOCNFs were chosen: 0.7 wt% and 0.9 wt%. The percentages of BW and glycerol were maintained at 10 wt% and 1 wt%, respectively. A smooth roll was chosen, the linear speed was set to 2 m min^−1^, and up to 7 mL of emulsion was applied per sheet. The coated sheets were first pre-dried using a thermoventilator blowing air at 50 °C for 60 s, and then left to dry at room temperature for 4 h. Shrinking effects were not observed.

Field-emission scanning electron microscopy (FE-SEM) was carried out to visualize the surface and cryosections of the paper sheets. Prior to that, the samples had been adhered to a sample holder using conductive tape and coated with carbon using a turbo evaporator from Emitech (Berlin, Germany), model K950. The electronic microscope employed was a Hitachi S-4100 device (Hitachi Ltd., Chiyoda, Japan) with a secondary electron detector and an acceleration voltage of 5 kV.

### 2.6. Characterization of BW-Coated Sheets

All the sheets were weighed, and their thickness was determined using a digital micrometer from Starrett (Athol, MA, USA). The static contact angle of water and castor oil for the uncoated and coated sheets was measured using a Krüss Scientific’s Drop Shape Analyzer, model DSA25B (Madrid, Spanish branch office) using the sessile drop method [26].

The water vapor transmission rate (WVTR) was estimated using the dry cup method [27]. Briefly, disk-shaped paper samples were cut and placed on impermeable cups above silica gel. The cups were sealed with an O-ring and stored in a climatic chamber at a temperature of 23 °C and a relative humidity of 50%. The WVTR was then calculated using the weight differences measured across 24 h:

(1) $$WVTR=∆w/\left(A\times t\right)$$

where *A* is the transmission area and Δ*w* is the increment in weight from the beginning of the experiment to time *t*.

To assess the grease resistance of the paper sheets, the TAPPI procedure T559, commonly known as the “Kit test”, was followed [28]. The number assigned to the most aggressive mixture of castor oil, toluene, and heptane that the paper probe resisted is referred to as the “Kit rating”.

The air resistance of the coated and uncoated sheets was estimated using the Gurley method following ISO standard 5636/5 [29]. This corresponds to the time required for 100 mL of air to pass through a 6.45 cm^2^ cross-sectional area with a pressure gradient of 1.2 kPa.

## 3. Results and Discussion

### 3.1. Pickering Stabilization: Macroscopic Features

**Figure 1:**
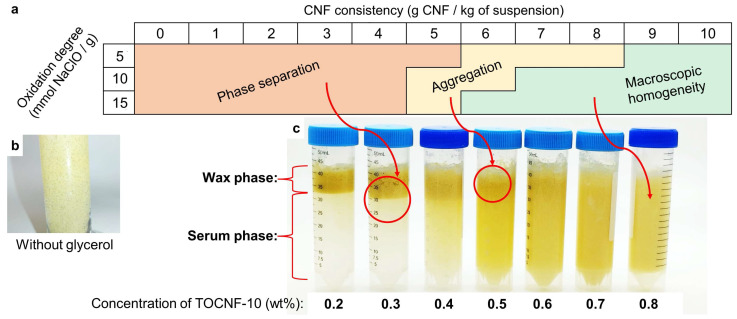
Macroscopic identification of phases at 23 °C based on the type and concentration of TOCNF (**a**), setting the proportion of BW at 10 wt% and that of glycerol at 1 wt%. The inset picture at the left (**b**) involves 0.6 wt% TOCNF-15, 10 wt%, and no glycerol. The inset picture at the right (**c**) correspond to samples with 1 wt% glycerol. Before photographying at a luminance of 310 cd m^−2^ and a color temperature of 6000 K, samples were left to settle for 24 h.

Below 0.5 wt% or 0.6 wt% of TOCNFs, depending on their degree of oxidation, the mixtures comprising 10 wt% BW, water, and CNFs quickly suffered from phase separation when undisturbed, regardless of the presence or absence of glycerol. More precisely, an aqueous or serum phase appeared at the bottom of the suspension, while the BW particles became aggregated as a layer in the upper part (“Phase separation” in Figure 1a). Keeping the BW proportion constant and increasing the concentration of TOCNFs resulted in an increase in the content of nanocellulose/water in the wax phase and/or an increase in the content of BW in the serum phase. This phenomenon is common among oil-in-water Pickering emulsions [30,31].

If the concentration was high enough, the interphase between the serum and wax became diffuse, but the BW could still be prone to creaming or beading, producing macroscopic particles that tended to be buoyant. This is labeled as “Aggregation” in Figure 1a. Finally, “Macroscopic homogeneity” implied a lack of visible beading, of particles visible to the naked eye, and of separation, even after at least three months of storage at 4 °C. The critical concentration to reach this homogeneity was 0.9 wt% in the case of TOCNF-5, 0.7 wt% in the case of TOCNF-10, and 0.6 wt% in the case of TOCNF-15. In general, this represents an advantage over nominally neutral nanocellulose, which usually requires a higher concentration to stabilize oil-in-water emulsions [32]. The higher the electrostatic repulsion between the nanofibers, the lower the critical concentration. In addition, when the proportion of the BW was increased to 20 wt%, attaining macroscopic homogeneity required at least 0.8 wt% TOCNF-15 or 1 wt% TOCNF-10.

It is worth noting that, as long as their concentrations were high enough, the TOCNFs sufficed to grant an even distribution of BW particles, avoiding phase separation. Nonetheless, these particles were macroscopic, unless glycerol was also incorporated into the system. This is shown in Figure 1b, for which the picture was taken with a flashlight for more evident visualization. In contrast, as illustrated by Figure 1c, in the presence of glycerol, the stabilization of microsized BW was possible, but only if the TOCNFs accounted for their critical concentration. Therefore, macroscopical homogeneity was a result of the synergistic effects of anionic nanocellulose and glycerol, in which the latter is suggested to work as softener or plasticizer.

The benefits of using glycerol in beeswax-containing systems have already been reported in other works, including as plasticizer for starch [13], as plasticizer for both chitosan and cellulose nanocrystals [33], and with only chitosan [34]. Nonetheless, the interactions between glycerol and BW, and not only between glycerol and the polysaccharide, are generally left unaddressed. However, glycerol is a well-known plasticizer for polyesters [35]; therefore, interactions between glycerol and the long-chain esters of BW, which would work as hydrogen bond acceptors, are plausible. This is further discussed when assessing optical microscopy images.

### 3.2. Assessment of Stable BW-In-Water Dispersions

**Figure 2:**
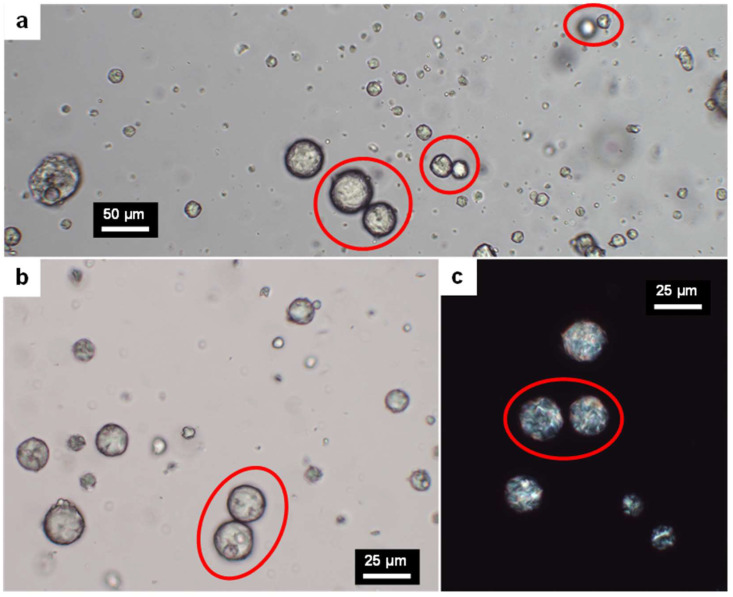
Optical microscopy images of 2-week-old beeswax-in-water dispersions stabilized with glycerol (1 wt%) and either 0.7 wt% TOCNF-10 (**a**) or 0.7 wt% TOCNF-15 (**b**). The latter system was also visualized under polarized light (**c**). Red-framed ellipses highlight doublets whose coalescence was avoided.

Although most of the BW particles dispersed in water were below 5 µm, the presence of large particles (more than 5 µm in diameter) that resisted buoyancy was attributed to physical hindrance. Figure 2 highlights doublets of these large particles that, even though they collided, did not coalesce. Despite this stabilization, there is little doubt that, if the continuous phase were solely water, the large particles would float due to a difference in density; i.e., the gravitational forces would outweigh Brownian motion [36,37]. Therefore, the continuous phase was characterized by a network of nanofibers that extended through the whole suspension, partially trapping water molecules between their hydration shells. The same reason has been adduced for other cases in which interfacial tensions do not provide a favorable driving force for the adsorption of TOCNFs on the dispersed phase [38]. This explanation does not depend on the degree of oxidation, since the dispersed phase was qualitatively similar in the cases of TOCNF-10 (Figure 2a) and TOCNF-15 (Figure 2b).

A recent review of ours dealt with the mechanisms by which anionic CNFs, such as TOCNFs, attain Pickering stabilization [1]. Applied to this case, it could be said that TOCNFs, due to their large hydration shell and its subsequent rheological effects, prevent phase separation by avoiding buoyance. In other words, the coalescence of BW droplets is not prevented by TOCNFs, but the fibrillar network they establish across the entire volume of the mixture hinders the movement of BW particles and compensates for gravitational effects. Nonetheless, in the presence of glycerol, there are favorable interactions between glycerol–nanofibers and glycerol–BW, preventing the coalescence of the latter even when its drops collide. The flexibility imparted by glycerol allows for lower angles between the tangent lines to the BW/water interface and the CNF/water interface (θ), respectively. The closer this angle, the higher the desorption energy (E_d_) [1]:

E_d_ = π R^2^ τ (1 + cos θ) (2)

where R is the Sauter’s diameter of BW droplets and τ is the BW/water interfacial tension. Hence, in the presence of glycerol, the adsorption/desorption equilibrium is shifted to the adsorption side. Once this condition is fulfilled, the energy barrier that two BW particles need to overcome for coalescence is due to the electrostatic repulsion between the equally charged (anionic) CNFs.

Figure 2c demonstrates that BW encompasses compounds with optical activity that are able to rotate the plane of polarized light. Chiral compounds that are known to be present in BW include hydroxy-monoesters and some branched hydrocarbons [39].

**Figure 3:**
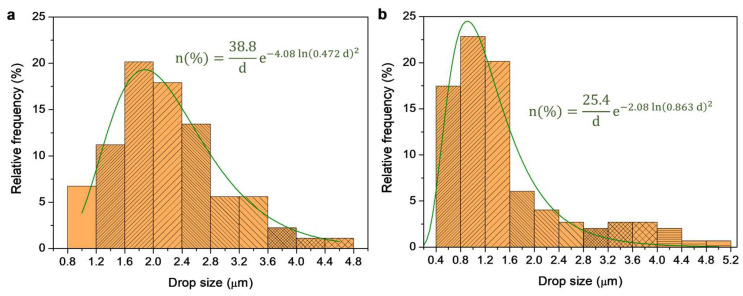
Size distribution of BW drops or particles whose size was equal to or lower than 5 ηm in emulsions stabilized either by TOCNF-10 (**a**) or TOCNF-15 (**b**). Fitting lines represent log-normal functions.

According to their size, there were different populations of BW particles. The size distributions in Figure 3 ignore particles with a Feret’s diameter >5 ηm, even though they comprised 15–20% of the total count or more than half of the total area. Nonetheless, the size of the large particles seemed to be randomly distributed. In contrast, small particles presented a non-random (albeit also non-normal) distribution that was significantly lopsided to the left (smaller size). The size distribution of BW/TOCNF-10/water and BW/TOCNF-15/water could be fitted to log-normal functions with adjusted correlation coefficients of 0.927 (Figure 3a) and 0.939 (Figure 3b), respectively.

As can be seen in Figure 3, the particle size attained using stabilization with TOCNF-15 was lower than that attained with TOCNF-10. In the latter case, drops in the 1.2–2.8 ηm range accounted for roughly 60% of the total population of particles. In the case of using TOCNF-15 as stabilizer, a similar percentage was comprised of particles in the 0.4–1.6 ηm range. In any case, it is worth remarking that, in terms of the area or volume, most of the emulsion consisted of large particles that were outside these distributions, as their Feret’s diameter adopted apparently non-adjustable values between 5 ηm and 40 ηm.

### 3.3. Rheology of BW/TOCNF/Glycerol/Water Systems

**Figure 4:**
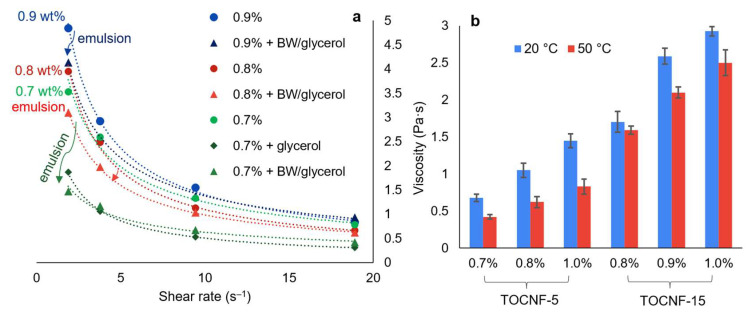
Apparent viscosity of BW-in-water emulsions compared with suspensions of the stabilizer (TOCNF-15) in water (**a**) and as a function of the degree of oxidation, TOCNF concentration, and temperature, displaying each factor at two levels and maintaining the shear rate at 19 s^−1^ (**b**). Error bars show twice the standard deviation.

Some relevant insights into the rheological behavior of TOCNF-stabilized BW-in-water emulsions are highlighted in Figure 4. With or without BW (and glycerol), the mixtures displayed shear-thinning behavior. Although the range of shear rates at which the apparent viscosity was measured corresponded to the pseudoplastic region, the overall behavior of the mixtures in the “macroscopically homogeneous” zone should be described as viscoplastic [40]. In other words, they flowed as long as their yield stress was exceeded, and further increasing the shear rate decreased their apparent viscosity. These features are common to all typical CNF suspensions and, in general, to CNF-stabilized oil-in-water emulsions [40,41].

Other generalities that were fulfilled included an increase in apparent viscosity with increasing TOCNF concentration (Figure 4a) and a decrease with an increasing temperature of the suspension (Figure 4b). Nevertheless, there were two phenomena whose explanation is not generic or self-evident: (i) the decrease in viscosity at a low shear rate and at 50 °C when incorporating BW, and (ii) the increase in viscosity with an increasing degree of oxidation.

The curves in Figure 4a correspond to a temperature of 50 °C, which is within a plausible range for paper coating. Seemingly, the more diluted the TOCNF suspension, the greater the decrease in the apparent viscosity when adding glycerol/BW. To a certain extent, this is due to the addition of 1 wt% glycerol. By itself, it is a practically Newtonian fluid and has a viscosity of ~0.9 mPa·s at 20 °C [42], but its plasticizer effect tended to decrease the viscosity of the TOCNF suspensions. Figure 4a shows an example corresponding to the system containing 0.7 wt% TOCNF-15. By hydrogen-bonding with glycerol, the TOCNFs were able to retain less water within their first and second solvation shells, which explains the high viscosity of anionic CNF suspensions at a low shear rate [1].

It is known that, if solvent–particle and particle–particle interactions are neglected, the addition of quasi-spherical solids to a colloidal system is expected to increase viscosity proportional to the volume fraction of solids [43]. Indeed, the effect of BW particles by themselves was either scarce, in the case of low TOCNF concentrations, or positive, thus compensating for the loss of viscosity that glycerol caused. Their influence on the shear-thinning behavior of TOCNFs was inconclusive, as this behavior was also observed in the emulsions. In the range of the shear rates that were analyzed, the apparent viscosity could be satisfactorily fitted (R^2^ > 0.98) to the following power law, widely known as the Ostwald-de Waele model [44]:

η = K × γ^n−1^ (3)

where γ is the shear rate, K is the consistency index, and n is the flow behavior index, with n < 1 indicating pseudoplastic or shear-thinning behavior. As can be seen from Table 3, BW/glycerol usually induced lower values of n, slightly enhancing the pseudoplastic character at shear rates of <20 s^−1^ at 50 °C. This claim should not be extended to other sets of conditions.

As for the positive influence of the degree of oxidation, this is partially due to the large hydration shells of the carboxylate groups, making TOCNFs retain more water, as previously described [1]. An additional reason lies in the lower size of BW droplets attained (Figure 3), implying a higher effective volume of the dispersed phase, even though the real volume fraction was the same regardless of the surface charge density. All considered, the emulsions stabilized with TOCNF-15 were selected for paper coating due to their higher ease of stabilization, higher viscosity attained for a given concentration, and a size distribution that favored smaller sizes.

### 3.4. Properties of BW-Coated Papers

**Figure 5:**
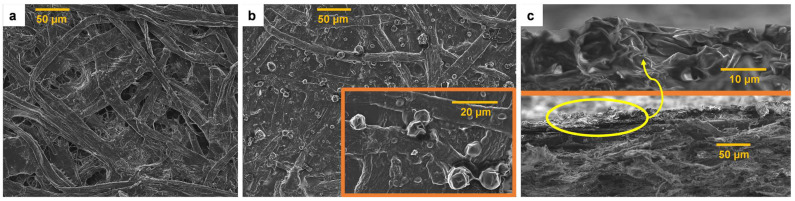
Micrographs of paper sheets: (**a**) surface of uncoated paper, (**b**) surface of a sheet coated with BW (10 wt%)/glycerol (1 wt%)/TOCNF-15 (0.9 wt%)/water, highlighting an inset image with a higher magnification; (**c**) cross-section of the latter sample at two levels of magnification.

Based on macroscopic observations, the coatings with BW-in-water emulsions yielded even distributions of each suspension onto the paper surface. The hydrophobic nature of BW eased drying, at least compared to nanocellulose-only coatings [45,46], and the sheets did not tend to shrink during the process. At a microscopic level, as shown in the SEM images of Figure 5, the BW particles retained the size that they had in the BW-in-water emulsions. While Figure 5a shows the surface of the original food-grade paper, two key differences can be observed in Figure 5b: first, the presence of BW particles that are mostly 1–5 µm in diameter; second, a more sealed, apparently less porous paper surface is visible.

The cross-section at Figure 5c allows us to appreciate the mostly amorphous morphology of a BW particle over paper. Rather than being an intrinsic characteristic of BW, its morphology is heavily dependent on the drying process. This globule-like shape that the BW particles adopted after drying differs from the more ordered, coral-like structure of other works where molten BW was allowed to cool down slowly [47] and crystallize.

The food packaging-grade paper had barrier properties that were significantly enhanced by the incorporation of BW and TOCNF-15. In terms of serving as a barrier to liquids, Table 4 demonstrates that the coated papers, although still unable to be deemed hydrophobic due to the relatively low water contact angles, retained a drop of water on their surface. In contrast, the original paper sheets absorbed the drop in less than 60 s. However, comparing these results with others of BW-based, nanocellulose-stabilized (albeit as nanocrystals) coating suspensions [48], the latter attained values over 110°. The weight gain of the paper in this case was higher than 15 g m^−2^, which could partially explain the difference.

The relatively narrow range of coat weights (Table 4) calculated based on the difference in weight between the coated and uncoated papers showed no significant correlation with the contact angle values (Pearson’s *r* = −0.10). Likewise, differences in coat thickness were mostly due to the drying process [49]. Hence, the failure to attain higher water contact angles was not due strictly to a low amount of BW on the paper, but rather to the proportion of highly hydrophilic components (glycerol, TOCNFs). In fact, in a side experiment, we increased the BW percentage to 20 wt% while keeping glycerol and TOCNF-15 at 1 wt% and 0.9 wt%, respectively, and this resulted in water contact angles of (115 ± 3)°. A micrograph of the surface in that case is displayed in Figure S1, showing a nearly complete coverage of the sheet with relatively large BW particles. In comparison, the coverage of the surface of paper by BW particles shown in Figure 5 seems incomplete and unevenly distributed, explaining the lower contact angles that were obtained. Furthermore, in another work using chitosan/glycerol as stabilizer and BW concentrations above 20 wt%, the authors attained water contact angles above 135° when applying the resulting emulsion to paper [24].

Interestingly, the emulsions improved not only the barrier properties to water, but also to grease. The oil contact angle increased from 20–30° to more than 60°, which should be attributed to the hydrophilic constituents of the emulsion (TOCNFs and glycerol) rather than to BW. Moreover, for the same reason, the coated papers attained a Kit rating of 5. In other words, they resisted a mixture of 60 vol% castor oil, 20 vol% heptane, and 20 vol% toluene, while the original papers only resisted pure castor oil. The enhancement in grease resistance due to the amphiphilic coating suspension was not fully expected. Nonetheless, Tyagi et al. [50] attained better results in this context using cellulose nanocrystals, montmorillonite, alkyl ketene dimer, and a protein compared to using nanocrystals alone. As they explained, interactions between the nanocellulose and hydrophobic moieties induced a favorable packing of the former due to the formation of cellulose–cellulose hydrogen bonds during drying.

**Figure 6:**
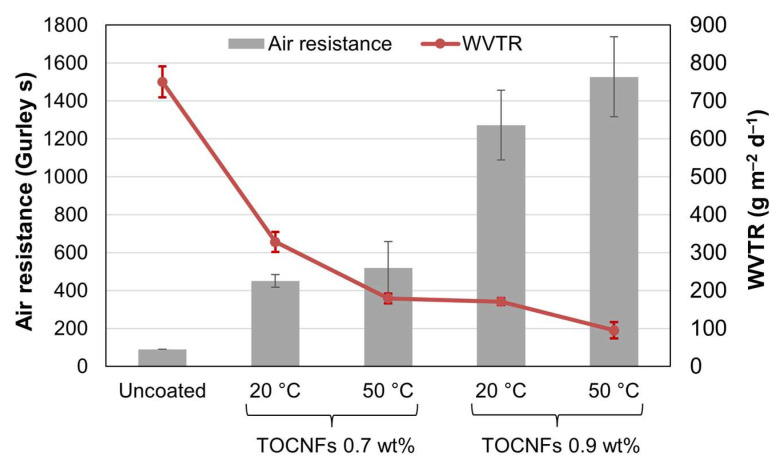
Gaseous barrier properties of BW-coated papers: air resistance (columns) and WVTR (line), depending on the concentration of stabilizer (TOCNF-15) and the temperature of the coating suspension. Error bars comprise twice the standard deviation.

Regarding the resistance to airflow and water vapor flow, both were greatly enhanced by coating with BW/glycerol/water mixtures, although generally falling short of conventional, commercially available polyethylene-laminated paper. As shown in Figure 6, the air resistance increased from 90 Gurley s to roughly 400–600 s when the mass percentage of TOCNFs was 0.7%, and then to 1100–1700 s when it was 0.9%. Given this dependence on the concentration of nanofibers, it is reasonable to state that this agent is the main contributor to the air barrier properties. Nonetheless, in previous works, coating suspensions consisting only of TOCNFs attained a lesser improvement [45,51]. Hence, glycerol played an important role, acting on nanofibers as a plasticizer and easing their even distribution onto paper.

While TOCNFs and glycerol were responsible for the enhancement in air resistance, the sharp decrease in WVTR (Figure 6) cannot be explained without BW. In the case of water vapor, all three components were relevant. On the one hand, TOCNFs and glycerol granted low porosity, as discussed above, thus impairing the kinetics of water vapor diffusion.

## 4. Conclusions

The ability of anionic cellulose nanofibers to stabilize beeswax-in-water systems seemingly arose from both electrostatic repulsion and rheological hindrance. Evidence for the first mechanism was found in the fact that the critical concentration to attain macroscopic homogeneity followed this trend: TOCNF15 < TOCNF10 < TOCNF5. At the same time, some particles were large enough (>10 ηm) to experience buoyancy if the rheological properties of the medium resembled those of water. However, buoyancy was prevented when undisturbed by the existence of yield stress or, when flowing, hindered by high viscosity. In fact, the apparent viscosity of TOCNF-stabilized emulsions was within the range of 0.5–2.5 Pa·s even at 50 °C and at 19 s^−1^, plausible temperature and a plausible shear rate conditions for paper coating, respectively. The degree of oxidation of TOCNFs was shown to exert a positive influence on the viscosity of the emulsions.

BW drops solidified as globule-shaped particles onto the surface of the paper. The incomplete covering of this surface implied that the water contact angles (~60–100°) were lower than those attained in other works using nanocellulose-stabilized BW (higher than 110°). However, while the resulting paper sheets were not as hydrophobic as intended, they resisted grease to a greater extent than expected, with a Kit rating of 5 and oil contact angles above 60°. Likewise, the barrier properties to air and water vapor were greatly improved over those of the original food-grade paper, albeit still not meeting the specifications of polyethylene-laminated paper.

## Figures and Tables

**Table 1:** Representative examples of the Pickering stabilization of BW-in-water emulsions involving polysaccharide-based materials in the literature.

Stabilizer	Concentration of Stabilizer	Concentration of Beeswax	Purpose	Ref.
Cellulose nanocrystals	0.03–0.15 wt%	1.5 wt%	Starch-supported films	[13]
Carboxymethyl chitosan with unmodified CNFs	1–5 wt%	10 vol%	Edible coating for food	[23]
Chitosan	1–3 wt%	10–30 wt%	Paper coating	[24]

**Table 2:** Key characteristics of TOCNFs with different degrees of oxidation.

Sample	Carboxyl Group Content (mmol/g)	Surface Charge Density (meq/g)
TOCNF-5	0.76 ± 0.04	−1.30 ± 0.00
TOCNF-10	1.11 ± 0.09	−1.64 ± 0.10
TOCNF-15	1.39 ± 0.10	−1.92 ± 0.16

**Table 3:** Ostwald–de Waele parameters of aqueous suspensions of TOCNF-15 at 50 °C and BW-in-water emulsions stabilized with TOCNF-15.

TOCNF-15 Concentration (wt%)	CNF Suspension		BW Emulsion	
	K (Pa s^n^)	n	K (Pa s^n^)	n
0.7	5.8 ± 0.4	0.66 ± 0.06	2.2 ± 0.2	0.55 ± 0.06
0.8	6.7 ± 0.3	0.79 ± 0.04	4.92 ± 0.08	0.70 ± 0.01
0.9	8.0 ± 0.2	0.76 ± 0.02	6.2 ± 0.1	0.65 ± 0.01
1.0	8.05 ± 0.09	0.72 ± 0.01	9.8 ± 0.3	0.84 ± 0.03

**Table 4:** Gains in basis weight and thickness after drying, water contact angle, and grease resistance of BW-coated papers, depending on the temperature of the suspension and on the mass percentage of TOCNFs.

Coating	T (°C)	Coat Weight (g m^−2^)	Coat Thickness (µm)	Static Contact Angle (°)		Kit Rating
				Water	Oil	
None	--	--	--	Drop absorbed	25.5 ± 5.0	1
10% BW, 1% glycerol	20	6.6 ± 1.9	5.9 ± 0.7	71.3 ± 0.5	65.5 ± 0.6	5
0.7% TOCNF-15	50	5.3 ± 1.1	8.5 ± 1.1	66.1 ± 9.6	64.8 ± 0.5	5
10% BW, 1% glycerol	20	8.3 ± 1.2	8.5 ± 1.5	79.8 ± 0.8	67.1 ± 4.4	5
0.9% TOCNF-15	50	5.2 ± 1.7	8.7 ± 1.8	96.1 ± 3.9	62.6 ± 0.7	5

## Data Availability

All data presented in the article are available upon request.

## Supplementary Materials

The following supporting information can be downloaded at: https://www.mdpi.com/article/10.3390/nano13162353/s1, Figure S1: SEM image of the surface of a sheet coated with BW (20 wt%)/glycerol (1 wt%)/TOCNF-15 (0.9 wt%)/water.
