# Supplementary figures and images for: Stabilization of Beeswax-In-Water Dispersions Using Anionic Cellulose Nanofibers and Their Application in Paper Coating
Source: Nanomaterials (Basel). 2023 Aug 16;13(16):2353. doi: 10.3390/nano13162353 (PMC10459156; doi:10.3390/nano13162353)

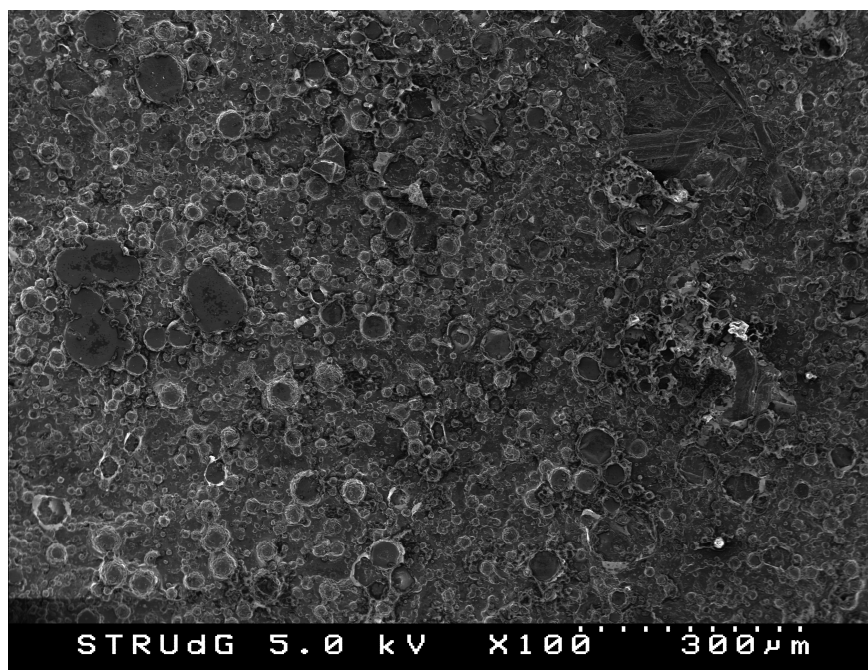

Figure S1: SEM image of the surface of a sheet coated with BW (20 wt%)/glycerol (1 wt%)/TOCNF-15 (0.9 wt%)/water

Supplement: Supplementary file 1 [file nanomaterials-13-02353-s001.zip › nanomaterials-2544910-supplementary.pdf]
